# A Comprehensive Study of Dietary and Lifestyle Correlates of Erythrocyte Membrane Fatty Acids and Oxidative Stress and Their Association with Maternal and Neonatal Outcomes

**DOI:** 10.3390/nu18172859

**Published:** 2026-09-01

**Authors:** Ksenija Nikolajeva, Anna Lece, Andrejs Šķesters, Vinita Cauce, Dzintars Začs, Laila Meija

**Affiliations:** 1 Riga East Clinical University Hospital, 2 Hipokrata Street, LV-1038 Riga, Latvia; laila.meija@rsu.lv; 2 Doctoral Department, Faculty of Medicine, Riga Stradins University, 16 Dzirciema Street, LV-1007 Riga, Latvia; 3 Department of Rehabilitation, Riga Stradins University, 26a Anninmuizas Boulevard, LV-1067 Riga, Latvia; 4 Institute of Occupational Safety and Environmental Health, Riga Stradins University, LV-1067 Riga, Latvia; anna.lece@rsu.lv (A.L.); andrejs.skesters@rsu.lv (A.Š.); 5 Statistics Unit, Riga Stradins University, 14 Balozu Street, LV-1048 Riga, Latvia; vinita.cauce@rsu.lv; 6 Scientific Institute of Food Safety, Animal Health, and Environment, Lejupes Street 3, LV-1076 Riga, Latvia; dzintars.zacs@bior.lv

**Keywords:** pregnancy, fatty acids, nutrition, oxidative stress

## Abstract

**Background:** Erythrocyte fatty acid (FA) and oxidative stress levels are influenced by a range of factors. The aim of this study was to evaluate erythrocyte FA profiles and oxidative stress markers and their relationships with each other, nutrition, lifestyle, gestational age, and pregnancy outcomes in pregnant Latvian women. **Methods:** A total of 74 pregnant and postpartum women were cross-sectionally examined. Dietary intake was assessed using a validated food frequency questionnaire; erythrocyte FA composition was analyzed using gas chromatography with a flame ionization detector; and oxidative status was assessed based on Ferric Reducing Antioxidant Power (FRAP) assays, glutathione peroxidase (GPx) activity, and selenoprotein P (SEPP1) and malondialdehyde (MDA) levels. **Results:** Dietary fats constituted 42.0% of the total caloric intake. Saturated fatty acids (SFAs) predominated in erythrocytes (55.7%), with the highest concentration observed for 16:0. Although multiple significant correlations between dietary factors and laboratory parameters were identified, none remained significant after correction for multiple comparisons. Erythrocyte 18:3, *n*-3 was significantly associated with FRAP (*p* < 0.05). Gestational complications and age did not affect biochemical parameters (*p* > 0.05), though gestational age correlated with MDA (r = 0.377, *p* < 0.05) and pre-pregnancy body mass index (BMI) with MUFAs (r = 0.373, *p* < 0.01). The FA composition differed depending on smoking status and alcohol consumption. **Conclusions:** Given the exploratory nature of this study, findings should be interpreted with appropriate caution. Several associations retained statistical significance following correction for multiple comparisons, namely between erythrocyte 18:3, *n*-3 and FRAP, alcohol consumption and smoking with erythrocyte fatty acid profiles, maternal age and plasma MDA concentrations, and pre-gestational BMI and MUFA levels. The remaining associations did not withstand correction and should therefore be regarded as hypothesis-generating, warranting confirmation in larger, adequately powered, confirmatory studies.

## 1. Introduction

During pregnancy, the maternal body undergoes many physiological changes to support fetal growth and prepare for childbirth [1]. Physiological adaptation is required to maintain maternal homeostatic balance [2,3].

Pregnancy is characterized by increased generation of reactive oxygen species (ROS), especially in the second half of gestation, due to increased basal metabolism, increased oxygen consumption, physiological insulin resistance, and activation of lipolysis [2,3]. The placenta is the main source of ROS due to its high energy demands and dynamic fluctuations in partial oxygen pressure [4]. Moderate ROS production is necessary for trophoblast differentiation, vascular remodeling, and angiogenesis via redox-dependent signaling pathways [4]. An imbalance between ROS generation and antioxidant defense results in oxidative stress, leading to oxidative modification of biomolecules, cell membrane damage, and induction of apoptosis [2,3]. Excessive oxidative stress is associated with gestational complications, including recurrent miscarriage, preeclampsia, intrauterine growth restriction, congenital malformations, and antenatal death [3,4].

Fetal development is largely dependent on maternal nutrient supply and placental integrity [5]. A healthy, nutrient-rich, and energy-balanced diet during pregnancy is critical for optimal fetal development and growth [6]. In turn, maternal malnutrition and antioxidant deficiency can induce oxidative stress [2].

Fatty acids (FAs) play a critical role in health by supporting a variety of physiological functions, including maintaining cell membrane integrity, promoting cell signaling, regulating cellular metabolism, and producing eicosanoids and cytokines. FAs and their derivatives, such as prostaglandins, play signaling roles that may influence pregnancy outcomes by regulating inflammatory responses [7,8]. FA levels in red blood cell (RBC) membranes have been proposed as objective indicators of past-120-day dietary FA intake and can serve as prognostic biomarkers for reproductive health outcomes and maternal–fetal complications [9,10].

Pregnancy-associated physiological changes drive substantial modifications in lipid metabolism, resulting in progressive elevations in total cholesterol, low-density lipoprotein cholesterol, and triglyceride concentrations, with a comparatively moderate increase in high-density lipoprotein cholesterol relative to pre-pregnancy baseline levels [1]. During pregnancy, FA metabolism undergoes substantial changes influenced by hormonal and physiological adaptations. Studies consistently report an overall increase in total FA concentrations across gestation, with total saturated fatty acids (SFAs), monounsaturated fatty acids (MUFAs), and *n*-6 polyunsaturated fatty acids (PUFAs) generally rising from the first to the third trimester, while long-chain polyunsaturated fatty acids (LCPUFAs), particularly arachidonic acid, eicosapentaenoic acid (EPA), and docosahexaenoic acid (DHA), demonstrate relatively smaller increases or even decline in later gestation, likely reflecting preferential placental transfer to the fetus and limited enzymatic conversion capacity [11,12,13,14,15,16]. Notably, the most substantial maternal free FA mobilization and placental FA transfer occur during the third trimester, when fetal demands are greatest [12].

Oxidative stress and erythrocyte membrane phospholipid FAs are related to various factors, including maternal diet, lifestyle, genetics, age, and maternal health status [2,10,17,18]. Despite the importance of oxidative stress, nutritional status, and lipid profiles during the gestational period, research in this area remains limited [9].

In this study, we aimed to assess FA indices in erythrocyte membrane phospholipids and oxidative stress indices in Latvian pregnant women and analyze their correlations with one another and with dietary and lifestyle factors, gestational age, pregnancy complications, and newborn anthropometric data.

## 2. Materials and Methods

### 2.1. Study Population

**Figure 1:**
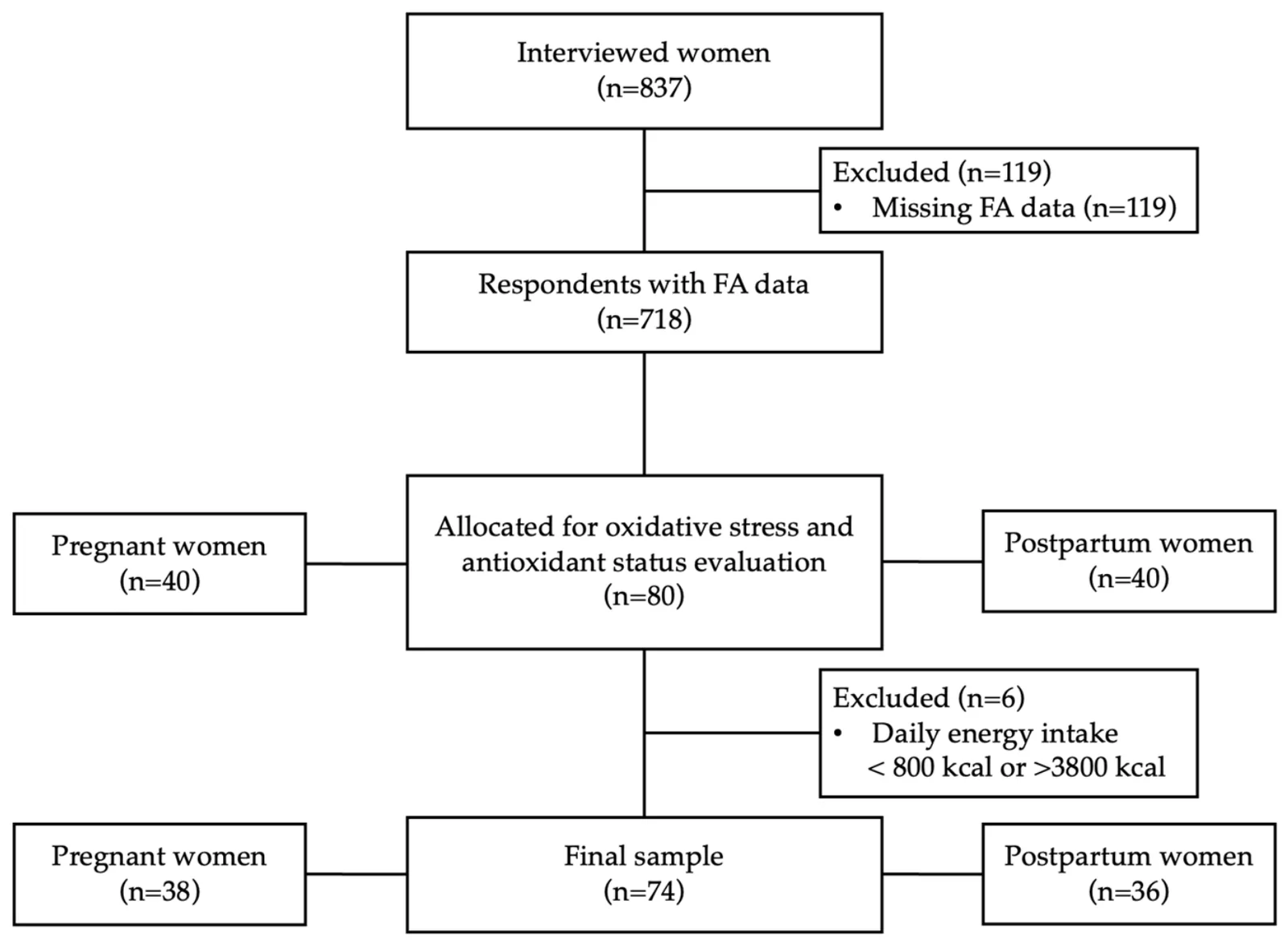
Schematic presentation of the sampling procedure.

This cross-sectional study was conducted as part of the Latvian Council of Science project “Excess Weight, Dietary Habits, and Vitamin D and Omega-3 Fatty Acid Status in Pregnancy,” project no. Izp-2019/1-0335, which included additional analysis of respondents’ blood plasma samples at the Institute of Occupational Safety and Environmental Health. During the project’s planning stage, quota sampling was used to stratify the target population by region of residence based on 2019 demographic data on reproductive-age women in Latvia, considering seasonal fluctuations. The reference population, as reported by the Central Statistical Office, comprised 296,354 women of reproductive age residing in Latvia in 2019, which constituted the basis for sample quota calculation. The final analytical sample consisted of 800 postpartum women and 200 women in the antenatal period. Data collection was conducted from July 2020 to June 2023 at outpatient medical institutions in Riga and inpatient maternity departments in other Latvian cities. We excluded women who resided outside Latvia, were minors, or had multiple pregnancies, diabetes mellitus, short bowel syndrome, gastrointestinal diseases (celiac disease, inflammatory bowel disease), or eating disorders. As part of the project, pregnant women and postpartum women up to 7 days post-delivery were interviewed. After signing informed consent forms, each participant was assigned a unique identification number. Data collection included questionnaires, medical record analysis, and biological samples for FA analysis. The sampling procedure is presented schematically in Figure 1.

This cross-sectional study examined multiple associations between dietary and lifestyle factors, erythrocyte membrane FA composition, and oxidative stress markers, and their relationships with maternal and neonatal outcomes. Given the exploratory nature of these analyses, no single association was prespecified as a primary endpoint; instead, an a priori power analysis was conducted using SPSS software, version 24.0, based on Fisher’s r-to-z transformation to estimate the minimum required sample size for detecting statistically significant Spearman correlations. Assuming a statistical power of 0.70, a two-sided significance level of α = 0.05, and an anticipated effect size of r = 0.300, the minimum required sample size was calculated to be 72 participants. The 70% threshold was chosen a priori as an acceptable, though conservative, power level for this exploratory cross-sectional design, in which a more conventional 80% power was not feasible given recruitment constraints. For the prespecified subgroups—pregnant women (*n* = 38) and postpartum women (*n* = 36)—power to detect r = 0.30 was substantially lower, at approximately 45% and 43%, respectively, with minimum detectable correlations of approximately r = 0.40 and r = 0.41. The overall sample size calculation therefore does not extend to, and cannot justify, correlation analyses conducted within these subgroups.

A combined sampling method was used, combining stratified and simple random sampling. The target population was stratified based on participant status (pregnant/postpartum women) and region of residence, after which simple random sampling was conducted within each stratum. A total of 80 participants underwent plasma antioxidant analysis at the Institute of Occupational Safety and Environmental Health, with the final sample including 74 women aged 20 to 41 years, including pregnant women between 27 and 40 weeks of gestation, as well as postpartum women up to 7 days post-delivery.

### 2.2. Data Collection

To assess dietary intake over the past six months, a validated food frequency questionnaire (FFQ), adapted from the Scientific Institutes of Food Safety, Animal Health, and Environment’s “BIOR” version, was used [19,20]. The FFQ included data on the frequency and volume of consumption of 211 food items, covering foods and beverages grouped into 20 food categories, as well as food additives. The “Photo Atlas of Food Products and Food Portions” was used to help determine food and drink portion sizes [21], and the survey was conducted by trained interviewers.

Participants with a daily energy intake of less than 800 kcal or more than 3800 kcal were excluded from the analysis.

In addition, a structured 73-question questionnaire was used to collect data on demographic characteristics, lifestyle, health status, and dietary habits and was used alongside information from medical records, including anthropometric measurements, laboratory data, gestational complications, and pregnancy history.

### 2.3. Blood Samples

Venous blood samples were collected in certified laboratories immediately after completion of the questionnaire for postpartum women and one week after completion of the survey for pregnant women. A 4 mL sample of blood was drawn into EDTA-containing tubes, which were gently inverted 5–10 times to ensure proper mixing with the anticoagulant. Samples were kept at 2–8 °C during processing. Initial processing included centrifugation at 1000–2000 rpm for 10 min at 4 °C to separate erythrocytes from EDTA-plasma. The supernatant (EDTA-plasma) was carefully transferred into clean tubes using a Pasteur pipette (Vacutest Kima S.R.L., Arzergrande, Italy). Erythrocytes were resuspended, aliquoted into 1 mL portions, and transferred into separate tubes without added liquid. Plasma and erythrocyte fractions were processed within 4 h of collection and stored at −20 °C for subsequent analysis. Frozen samples were then transported to the Scientific Research Institute of Food Safety, Animal Health and Environment “BIOR” for erythrocyte FA analysis within 13 days of collection. Laboratory results were returned to pregnant participants in electronic form, with personal data privacy ensured by assigning participants individual identification codes. After determining FA composition, the plasma samples were frozen and transported to a branch of the Central Laboratory, where they were stored at −80 °C. The determination of oxidative stress parameters was carried out two years later, after the completion of the project: the samples were delivered to the Institute of Occupational Safety and Environmental Health under temperature-regime transportation and stored until analysis.

### 2.4. Measurement of FA Composition in Erythrocytes

FA analysis of erythrocyte membrane phospholipids was performed at the BIOR laboratory using gas chromatography with flame ionization detection (GC-FID). Frozen blood samples were removed from the freezer and allowed to thaw. After thorough mixing, a 200 µL aliquot of RBC mass was transferred to a separate tube. Then, 800 µL of distilled water was added to the resulting volume of RBC mass, followed by centrifugation for 10 min at 3000 rpm. The precipitate, representing the phospholipid membranes, was washed twice with 800 µL of distilled water and then centrifuged again to isolate the RBC membrane fraction. A total of 400 µL of distilled water and 3 mL of a 1:1 mixture of chloroform and methanol were added to the sediment and mixed thoroughly. The chloroform fraction was collected and transferred to a clean test tube, after which the solvent was removed by evaporation. Hydrolysis and methylation of phospholipids were performed simultaneously: 100 µL of butylated hydroxytoluene and 500 µL of boron trifluoride–methanol reagent were added to the sample, and the mixture was incubated for 60 min at 100 °C in hermetically sealed test tubes in a thermal block. After incubation and cooling, 800 µL of distilled water and hexane were added to extract FA methyl esters. The hexane layer containing the target compounds was transferred to a glass vial, evaporated to dryness under a stream of nitrogen, and reconstituted in 100 µL of hexane before chromatographic analysis. FAs were separated and detected using an Agilent 6890 N gas chromatograph (Agilent Technologies, Santa Clara, CA, USA) equipped with a flame ionization detector. Chromatographic data were processed using Agilent ChemStation software OpenLab CDS 3.0. FA methyl esters were identified by comparing their retention times with those of authentic FA methyl ester standards, including Supelco 37 Component FAME Mix (Supelco, Bellefonte, PA, USA). Retention time stability and instrument performance were verified within each analytical batch using calibration standards and quality-control samples.

Analytical quality control included reagent blanks, calibration/retention time standards, duplicate analyses, and pooled erythrocyte quality-control samples analyzed within and between batches. The method showed intra-assay coefficients of variation of 3–5% and inter-assay coefficients of variation of 3–6% for the major FAs. Analytical recoveries, assessed using spiked quality-control samples and/or internal-standard recovery, ranged from 93 to 108%. Limits of detection were determined according to the laboratory validation procedure and were set at 0.1 g/100 g lipids. Samples not meeting internal-standard recovery, retention time, blank, or quality-control acceptance criteria were reanalyzed or excluded according to the laboratory quality-assurance procedure.

The quantitative content of each FA was calculated by absolute calibration and expressed as a relative percentage of the total identified FA pool in each sample, calculated from the normalized GC-FID peak areas.

### 2.5. Measurement of Oxidative Stress and Antioxidant Status Markers

The samples were delivered to the Scientific Laboratory of Biochemistry at the Institute of Occupational Safety and Environmental Health, Riga Stradins University, under temperature-controlled transport conditions and were stored at −80 °C in an ultra-low temperature freezer (U 101 Innova, New Brunswick, Eppendorf AG, Hamburg, Germany; temperature range: −50 °C to −86 °C) until analysis. Prior to analysis, samples were thawed once and centrifuged at 3500× *g* for 10 min (refrigerated centrifuge 5804R, with swing-bucket rotor 4500 rpm, Eppendorf AG, Hamburg, Germany) and subsequently diluted, if required by the protocol. For MDA quantification, centrifugation was performed using a HERMLE Z 287 A centrifuge (maximum speed: 14,000 rpm [16,085× *g*], LaborTechnik, Wehingen, Germany).

The concentration of selenoprotein P (SEPP1) in plasma was determined using a Human SEPP1 ELISA kit, Cat No CSBEL 021018 HU, CUSABIO BIOTECH CO, Ltd., Wuhan, China, based on the sandwich ELISA principle, according to the manufacturer’s instructions. The SEPP1 concentration was calculated according to the calibration curve and expressed in mg/L. Plasma malondialdehyde (MDA) concentrations were determined using the thiobarbituric acid reactive substances (TBARS) method—OxiSelect™ TBARS (MDA Quantitation) Assay kit, Cat No STA-330, Cell Biolabs, Inc., San Diego, CA, USA—according to the manufacturer’s assay protocol [22]. All samples were analyzed in duplicate; hemolyzed plasma samples were not analyzed.

For Ferric Reducing Antioxidant Power (FRAP) assays, Cell Biolabs’ OxiSelect™ Ferric Reducing Antioxidant Power (FRAP) commercial kit was used according to the manufacturer’s protocol. Room-temperature 0.1 mL thawed plasma samples were pipetted into each microplate well, then 0.1 mL of Reaction Reagent was added, and the samples were incubated at 37 °C on a horizontal shaker for 10 min. The measurements were read at a wavelength of 593 nm using a multimodal microplate reader (SPARK, TECAN, Grödig, Austria), and the results were expressed in Fe^2+^ mmol/L.

Plasma glutathione peroxidase (GPx) activity was measured using the RANSEL kit (Randox Laboratories, Crumlin, UK). Prior to analysis, samples were diluted with the provided diluting agent, followed by the addition of Drabkin’s reagent to inhibit endogenous peroxidases and prevent falsely elevated results. The assay was performed at 37 °C, with GPx activity expressed in U/mL.

### 2.6. Ethics

This study was approved by the Clinical Research Ethics Committee of Riga Stradins University (No. 6-1/02/62). Prior to inclusion, participants were provided with written information about the study’s objectives and procedures. After obtaining informed consent, each participant was assigned a coded identification number for registration and subsequent data processing.

### 2.7. Data Analysis

Dietary intake data obtained from the food frequency questionnaire were analyzed at the Scientific Institute of Food Safety, Animal Health and Environment “BIOR” using a custom-developed software application based on Microsoft Dynamics AX 2009. This analytical tool operates in conjunction with the “BIOR” Food Composition Database, originally developed for the “Food Consumption Study of the Latvian Population (2012–2013)” and subsequently utilized in ongoing national dietary surveys. The database is derived from the German Max Rubner Institute’s food composition data and has been adapted to include Latvian-specific food products and traditional recipes. The dietary analysis provided detailed estimates of total energy intake, nutrients, vitamins, minerals, and FAs.

Statistical analysis was conducted using SPSS software, version 24.0. Descriptive statistical values—means, medians, standard deviations, interquartile ranges (Q1–Q3), and minimum and maximum values—were calculated to summarize and characterize the data distribution. Depending on the data distribution, intergroup differences were assessed using the Mann–Whitney test or *t*-test for two groups and the Kruskal–Wallis test for three or more groups. Correlation analysis was performed using Spearman’s rank correlation coefficient. A *p* value < 0.05 was considered statistically significant.

To evaluate the influence of maternal status on the identified associations, a sensitivity analysis was conducted in which correlations significant in the full cohort (*n* = 74), together with variables previously shown to differ between pregnant and non-pregnant participants using the Mann–Whitney U test, were re-assessed in pregnant participants alone (*n* = 38) using Spearman’s rank correlation coefficient. 95% confidence intervals were derived via Fisher’s r-to-z transformation, with standard errors computed according to Bonett and Wright.

Benjamini–Hochberg correction was applied to associations between laboratory parameters and dietary and lifestyle factors, whereas Holm-Bonferroni correction was used for correlations between erythrocyte FA parameters, markers of oxidative stress and antioxidant status, and neonatal anthropometric measurements. Residual-based partial Spearman correlation analyses were performed to evaluate the association between neonatal measurements (X) and FA and oxidative stress markers (Y), while adjusting for gestational age, maternal pre-pregnancy body mass index (BMI), and smoking status (Z). Gestational age and pre-pregnancy BMI were standardized to *z*-scores before inclusion as covariates in the regression models. The residual-based procedure was implemented by fitting separate linear regression models of X on Z and Y on Z using the original (untransformed) data. Residuals from these models, representing the variation in X and Y not explained by the covariates, were then extracted. Spearman’s rank correlation coefficient was calculated between the residuals to estimate the adjusted monotonic association. All statistical tests were two-sided, and a *p* value < 0.05 was considered statistically significant.

## 3. Results

### 3.1. Baseline Characteristics

Respondents’ baseline characteristics are shown in Table 1. The study involved women aged 20 to 41 years, and participants’ mean age was 31.0 ± 4.9 years. Of the pregnant women recruited, 44.6% (*n* = 33) were primiparous. Among postpartum women, 91.7% (*n* = 33) of newborns were delivered vaginally, with mean delivery at 39.1 ± 3.0 gestational weeks. Pregnant women were interviewed at 32.8 ± 3.0 weeks of pregnancy.

Of the respondents who reported alcohol consumption during pregnancy (*n* = 23), 82.6% (*n* = 19) reported this practice during the first trimester. Although 90.6% (*n* = 67) of the participants did not smoke during the gestational period, 70.3% (*n* = 52) reported a positive smoking history, with at least one year of smoking before conception. In addition, 44.6% (*n* = 33) of respondents reported adopting healthier dietary patterns during pregnancy.

Table 2 illustrates newborns’ anthropometric measurements. The mean APGAR score, used to assess physiological parameters, was 7.9 ± 0 (*n* = 34) at the first minute and 9.1 ± 0.4 after 5 min (*n* = 31).

### 3.2. Energy and Nutrient Intake

Respondents’ average energy and nutrient intakes are shown in Table 3. Dietary fat and SFAs made up 42.0% and 6.8% of total energy intake, respectively. The median dietary EPA and DHA intakes were 195.1 (126.1–318.0) mg and 231.2 (124.0–456.0) mg, respectively.

Respondents consumed FAs across different food groups, with FA intake during the study summarized in Table 4.

Table 5 represents nutrient intake from food supplements, where 29.7% (*n* = 22) of respondents used multivitamin complexes at the time of interview. EPA and DHA were consumed via multivitamin complexes and fish oil supplements.

### 3.3. Laboratory Parameters

Data on FA composition in erythrocyte phospholipids are summarized in Table 6. SFAs represented the predominant fraction of total FAs, with 16:0 found in the highest concentrations. When FA levels were compared between pregnant and postpartum participants, significantly lower DHA levels were found in postpartum women. In contrast, the pospartum group presented significantly higher levels of 20:2 (*n*-6) and SFAs.

The distribution of markers of oxidative stress and antioxidant status is shown in Table 7. No statistically significant intergroup differences were identified.

### 3.4. Associations Between Laboratory Parameters, Dietary and Lifestyle Factors, and Maternal and Neonatal Outcomes

**Figure 2:**
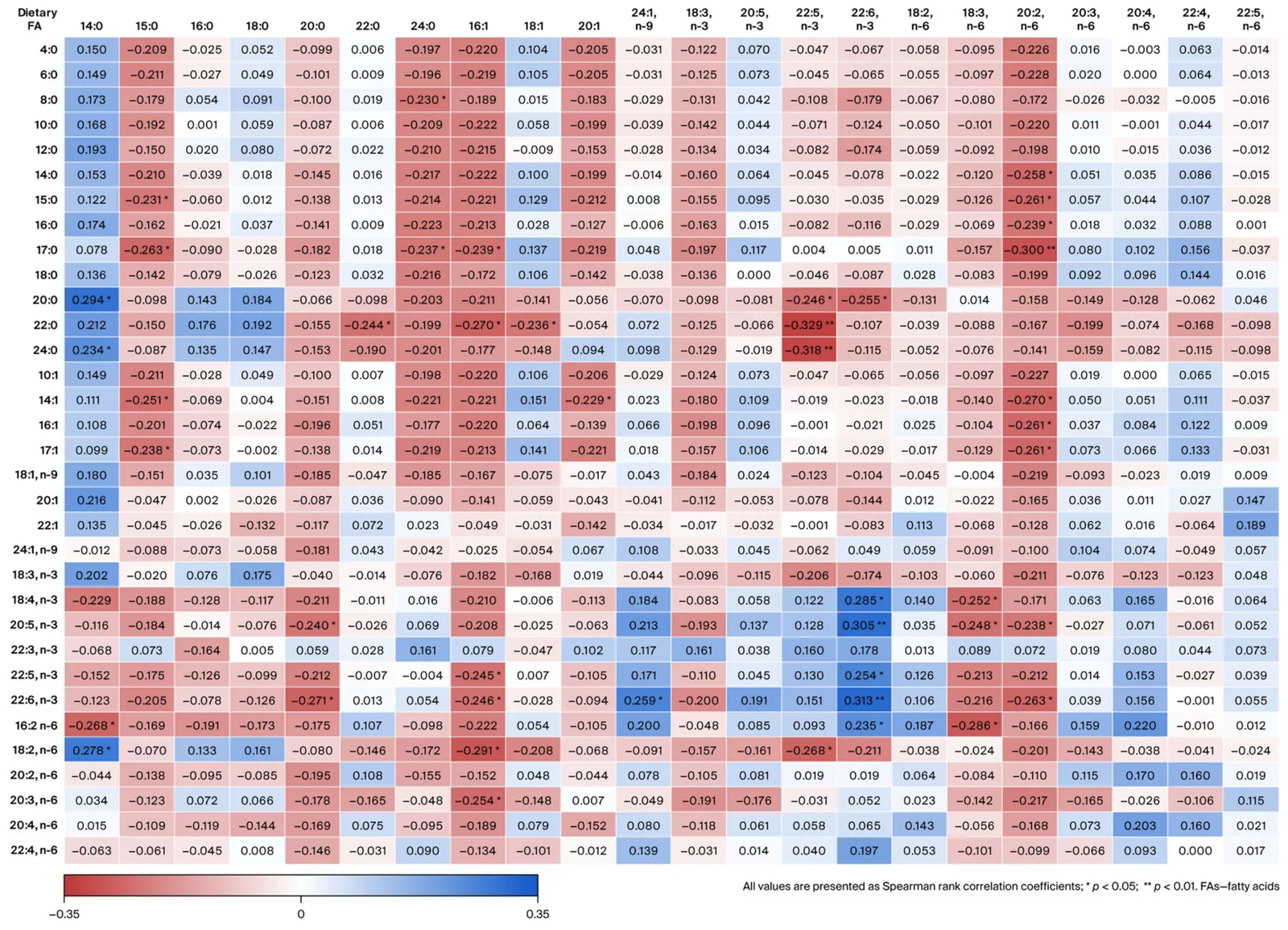
Correlations between dietary FAs and their concentrations in erythrocyte membrane phospholipids.

We found weak but statistically significant negative correlations between erythrocyte 20:2 (*n*-6) and dietary protein, fat, and soluble fiber levels, with r = −0.242, −0.245, and −0.237 (*p* < 0.05), respectively. Dietary carbohydrates and insoluble fiber positively correlated with erythrocyte 14:0, with r = 0.270 and 0.237, *p* < 0.05. A negative correlation was detected between dietary protein and 16:1, with r = −0.231 and *p* < 0.05. Many statistically significant negative correlations were found between erythrocyte 20:2 (*n*-6) and dietary FAs, including SFAs (14:0, 15:0, 16:0, and 17:0), MUFAs (14:1, 16:1, and 17:1), and *n*-3 PUFAs (20:5 and 22:6). Various negative correlations were also found with erythrocyte MUFA 16:1, notably 17:0, 22:0, *n*-3 PUFAs 22:5 and 22:6, and *n*-6 PUFAs 18:2 and 20:3. Multiple statistically significant positive correlations were identified for erythrocyte *n*-3 PUFA 22:6, such as *n*-3 PUFAs 18:4, 20:5, and 22:5, alongside a negative correlation with *n*-3 PUFA 20:0. Erythrocyte 22:5, *n*-3, was statistically negatively correlated with dietary SFAs (20:0, 22:0, and 24:0), as well as with *n*-6 PUFA 18:2, while dietary FAs 15:0, 17:0, 14:1, and 17:1 showed several significant positive correlations with erythrocyte 15:0. Dietary FA levels also demonstrated significant correlations with erythrocyte SFA 14:0, with notable associations including 20:0, 24:0, and *n*-6 PUFAs 16:2 and 18:2. Figure 2 presents all associations between dietary FA intake and erythrocyte membrane phospholipid FA composition. However, none of the correlations remained statistically significant after Benjamini–Hochberg correction for multiple comparisons.

When analyzing correlations between nutritional factors and FA concentrations in erythrocyte membrane phospholipids, statistically significant negative correlations were found between 20:2 (*n*-6) and vitamin A (r = −0.256; *p* < 0.05), vitamin K (r = −0.321; *p* < 0.05), and vitamin C (r = −0.230; *p* < 0.05). In addition, dietary iron had positive associations with 20:1 (r = 0.265; *p* < 0.05) and 15:0 (r = 0.244; *p* < 0.05) and a negative correlation with DHA levels, with r = −0.253 and significance at the *p* < 0.05 level. Erythrocyte 22:5, *n*-6 was negatively correlated with vitamin E (r = −0.233; *p* < 0.05), which was presented as a dietary equivalent of tocopherol. A negative correlation was also observed between dietary vitamin K and SFA 20:0 (r = −0.250; *p* < 0.05). Summarizing these associations between FA groups and dietary factors, a weak, negative, statistically significant correlation was identified between *n*-6 FAs and insoluble fiber (r = −0.246; *p* < 0.05). Table 8 shows associations between different dietary factors and markers of oxidative stress and antioxidant status. Statistically significant associations were found between dietary vitamin C and SEPP1, with r = −0.249 and *p* < 0.05, and between fructose and GPx, with r = −0.231 and *p* < 0.05. Following Benjamini–Hochberg adjustment for multiple comparisons, no statistically significant correlations were identified between nutrient intake and markers of oxidative stress and antioxidant status or erythrocyte FA levels.

No correlations between groups of dietary FAs and oxidative stress and antioxidant status markers were detected. Plasma MDA was negatively correlated with dietary 12:0 (r = −0.247, *p* < 0.05), 22:1, *n*-9 (r = −0.241, *p* < 0.05) and 8:0 (r = −0.240, *p* < 0.05), while dietary 22:0 levels were weakly positively correlated with plasma SEPP1 (r = 0.249, *p* < 0.05); however, none of these correlations remained statistically significant after adjustment. Correlations between erythrocyte FAs and oxidative stress markers are shown in Table 9. In the unadjusted analyses, positive correlations were observed between erythrocyte 16:1 (r_s_ = 0.258, *p* = 0.029, 95% CI: 0.024–0.465), 18:3, *n*-3 (r_s_ = 0.359, *p* = 0.002, 95% CI: 0.132–0.551), 18:3 *n*-6 (r_s_ = 0.244, *p* = 0.039, 95% CI: 0.009–0.453), and 20:2, *n*-6 (r_s_ = 0.291, *p* = 0.013, 95% CI: 0.059–0.493) with FRAP. A weak positive correlation was also found between erythrocyte 18:3, *n*-3 and GPx activity (r_s_ = 0.232, *p* = 0.047, 95% CI: 0.000–0.440). After adjustment for maternal status using residual-based partial Spearman correlation, the associations between FRAP and erythrocyte 16:1 (r_s_ = 0.280, *p* = 0.017, 95% CI: 0.048–0.484), 18:3, *n*-3 (r_s_ = 0.337, *p* = 0.004, 95% CI: 0.108–0.532), 18:3, *n*-6 (r_s_ = 0.239, *p* = 0.043, 95% CI: 0.005–0.449), and 20:2, *n*-6 (r_s_ = 0.284, *p* = 0.016, 95% CI: 0.051–0.487) remained statistically significant. The correlation between erythrocyte 18:3, *n*-3 and GPx was slightly attenuated but remained of borderline statistical significance (r_s_ = 0.229, *p* = 0.050, 95% CI: −0.003 to 0.437). Only the correlation between 18:3, *n*-3 and FRAP remained statistically significant after Benjamini–Hochberg adjustment.

In a sensitivity analysis restricted to pregnant participants (n = 38), correlations between oxidative stress markers (FRAP and GPx) and individual FAs largely lost statistical significance, although the direction of the associations remained unchanged. Specifically, the correlation between FRAP and 18:3, *n*-3 decreased from r = 0.359 (*p* = 0.002) to r = 0.271 (*p* = 0.105); FRAP and 18:3, *n*-6 decreased from r = 0.244 (*p* = 0.039) to r = 0.224 (*p* = 0.183); and GPx and 18:3, *n*-3 decreased from r = 0.232 (*p* = 0.047) to r = 0.113 (*p* = 0.500). The correlation between FRAP and 20:2, *n*-6 showed a similar pattern, moving from r = 0.291 (*p* = 0.013) to r = 0.322 (*p* = 0.052). The only oxidative stress–FA association that retained statistical significance in the pregnant subgroup was FRAP and 16:1, *n*-9 (r = 0.258, *p* = 0.029 in the full sample vs. r = 0.356, *p* = 0.030 in pregnant participants). In contrast, correlations among FAs themselves—particularly those involving 22:6, *n*-3—all remained statistically significant in the pregnant subgroup, and for most the absolute strength of the correlation increased relative to the full sample: 20:4, *n*-6 and 22:6, *n*-3 (r = 0.771 to *p* = 0.829), 20:0 and 22:6, *n*-3 (r = −0.335 to r = −0.579), 22:0 and 22:6, *n*-3 (r = 0.324 to r = 0.409), 20:2, *n*-6 and 22:6, *n*-3 (r = −0.387 to r = −0.429), 20:1, *n*-9 and 22:6, *n*-3 (r = −0.350 to r = −0.417), and 18:3, *n*-3 and 22:6, *n*-3 (r = −0.251 to r = −0.372).

Differences in erythrocyte phospholipid FA composition among respondents with different smoking and alcohol use behaviors and physical activity levels are summarized in Table 10. Statistically significant differences in oxidative stress and antioxidant parameters among participants with differing smoking habits, alcohol consumption patterns, and levels of physical activity were not detected.

In our sample, FA composition, as well as oxidative stress and antioxidant status, in erythrocyte phospholipids did not differ significantly between groups with and without gestational complications (*p* > 0.05). Analysis of the relationship between the gestational week at the time of delivery and levels of SFAs, MUFAs, PUFAs, and individual FAs in erythrocyte membrane phospholipids revealed no significant correlations, though gestational age at delivery was statistically significantly correlated with plasma MDA, with r = 0.377 and *p* = 0.026. No significant differences were observed in oxidative and antioxidative marker levels in respondents in different BMI groups, though BMI correlated with erythrocyte MUFAs, with r = 0.373 and *p* < 0.01, and a statistically significant difference was detected in 18:1 levels among groups with different BMI levels. However, no difference was found in subgroup analyses (*p* > 0.05).

Table 11 summarizes the associations between erythrocyte FAs, markers of oxidative stress and antioxidant status, and newborn anthropometric measurements. Nominally significant correlations were identified between newborn head and chest circumferences and erythrocyte SFAs, MUFAs, PUFAs, *n*-6, and FRAP. Newborn weight was negatively correlated with erythrocyte *n*-6 (r = −0.393, 95% CI: −0.647 to −0.062, *p* = 0.018, *p_adj_* = 0.144) and PUFAs (r = −0.339, 95% CI: −0.607 to −0.002, *p* = 0.043, *p_adj_* = 0.301) and was positively correlated with FRAP (r = 0.411, 95% CI: 0.076–0.663, ESS = 36, *p* = 0.014, *p_adj_* = 0.126). Newborn height showed a positive correlation with FRAP (r = 0.347, 95% CI: 0.000–0.620, ESS = 35, *p* = 0.044, *p_adj_* = 0.396). After applying the cumulative-max rule, none of the hypotheses remained statistically significant following Holm–Bonferroni correction at the 0.05 level. As shown in Table 12, after adjustment for gestational age, pre-pregnancy BMI, and maternal smoking status (ESS = *n* − 3 in each case), residual-based partial Spearman correlations revealed nominally significant associations between FRAP and both newborn weight (r = 0.369, 95% CI: 0.017–0.639, ESS = 33, *p* = 0.035) and head circumference (r = 0.477, 95% CI: 0.134–0.718, ESS = 32, *p* = 0.006); however, neither association remained significant after Holm–Bonferroni correction (*p_adj_* = 0.315 and *p_adj_* = 0.054, respectively).

## 4. Discussion

Nutrition during pregnancy is critical for both mother and fetus, as both over- and undernutrition significantly impact neonatal health and pregnancy outcomes [23]. Metabolic programming begins in utero, and the first 1000 days after conception determine the future health of the offspring [24,25]—a paradigm known as the Developmental Origins of Health and Disease (DOHaD) [26]. We compared our results with data from other cohorts [23,24]. Across 33 cohort studies (*n* = 135,566), the mean protein, fat, and carbohydrate intakes were 78.21 g, 74.17 and 262.17 g, respectively [23], whereas our respondents demonstrated higher protein and fat but lower carbohydrate intakes of 102.0 g, 104.0 g, and 215.5 g per day, respectively. Although the proportion of fat intake in total energy intake (42.0%) exceeded the levels recommended by the Latvian Ministry of Health and WHO Europe (<30%) [27], a similar trend was also observed in other European countries [28].

The composition of the mother’s diet affects the transfer of FAs to the fetus, while disruption of lipid metabolism increases fat accumulation in newborns and predisposes them to obesity and metabolic diseases [26]. Although SFAs are necessary for energy storage, their excess leads to blood clotting, increased cholesterol levels, and insulin resistance, contributing to gestational hyperglycemia [26,29]. In our study, dietary SFAs were found in higher amounts than in women from the mother–child birth cohort PreventADALL (*n* = 1674)—16.7 (12.0–23.0) g vs. 12.5 (10.9–14.1) g—but did not exceed the recommended limit of 10% of TEE [6,30]. The predominant dietary FAs were MUFAs, accounting for 21.5% of total energy expenditure. Omega-6 FA consumption was specified as 5–10% of TEE, and respondents met this recommendation, with an omega-6 intake of 16.7 g per day, or 5.3% of TEE [31,32,33]. DHA consumption also reached the recommended 200–300 mg/day [27], higher than the typical Western values of 70–200 mg/day [34]; EPA and DHA intakes were 0.2 (0.1–0.3) and 0.2 (0.1–0.5) g/day, respectively, with other researchers reporting intakes of 0.1–0.2 and 0.2–0.3 g/day, respectively [35,36]. Among dietary PUFAs, LA, an *n*-6 FA, was identified at the highest level, corresponding with data from other studies, as did ALA levels [35,36]. The main sources of MUFAs and SFAs were fats and oils and milk and dairy products, respectively, while PUFAs were consumed mainly via fats, oils, and meat and meat products, with the latter containing exclusively *n*-6 FAs. These findings align with those of a French study that identified vegetable oils and butter as the main sources of these fats [36].

Physiological reactive oxygen species (ROS) levels play an important regulatory role in reproductive processes, including folliculogenesis, oocyte maturation, embryogenesis, implantation, and fetoplacental development [37]. Oxidative stress, characterized by excessive ROS generation, has adverse effects on pregnancy, leading to implantation failure, miscarriage, pre-term birth, low birth weight, and malformations due to placental dysfunction [38]. Studies confirm the presence of oxidative stress markers in various pregnancy complications, including preeclampsia, intrauterine growth restriction, and gestational diabetes, which are characterized by increased MDA and decreased GPx activity [39,40,41,42]. Comparison with other studies showed that FRAP levels were 286.8 ± 52.3 µmol/L and 281.5 (240.0—324.8) µmol/L in the second and third trimester, respectively, in uncomplicated pregnancies (*n* = 30), which were lower than our median values [43]. The mean MDA level in healthy pregnant women was 1.07 ± 0.37 mmol/L, which was also lower than our result of 4.0 (2.7–5.3) mmol/L [44].

The role of antioxidant vitamins remains controversial. Vitamin C, despite its predominantly antioxidant effect, can exhibit prooxidant properties in the presence of iron excess, as evidenced by the positive correlation between its intake and MDA levels [45].

FAs play a key role in fetal and placental development, mediating cell signaling, proliferation, and differentiation. During early placentogenesis, they promote trophoblast angiogenesis, and their deficiency can impair placental function and lead to obstetric complications. During the third trimester, the fetus’s need for long-chain polyunsaturated fatty acids (LCPUFAs) increases significantly, making the FA composition of maternal RBC an important factor in fetoplacental development and pregnancy outcome [10,46]. In our study, SFAs were the dominant fraction in RBC, accounting for more than half of their total content—a result consistent with data from other authors [47,48]. Total MUFAs were present at higher concentrations compared with pregnant women from Chile, China, and Belgium—15.6% vs. 13.3%, 14.5% and 12.7%, respectively [48]. Among individual erythrocyte FAs, 16:0 accounted for the largest proportion, which is consistent with the findings of other studies [15,48]. It should be noted that there are few studies of FA profiles in healthy pregnant women, as most focus primarily on *n*-3 PUFAs, and they are difficult to compare due to differences in the biological material used [12,35,48,49,50]. The concentration of 18:1 *n*-9 was comparable to that reported in maternal erythrocytes of Japanese women (n = 74) [15]. According to data from a birth cohort (*n* = 607), the median content of *n*-3 and *n*-6 PUFAs in maternal erythrocytes was 9.69% and 36.33%, respectively [51], which exceeds the values of our respondents—7.2% and 14.8%. Moreover, among individual PUFAs, we observed the highest content for linoleic acid, which is at odds with several other publications [51]. In addition, the study population exhibited lower levels of 18:2, *n*-6 and 20:4, *n*-6 compared with cohorts from other countries [48]. DHA and EPA, critically important for neurogenesis, neural connection formation, and protection against oxidative stress, are associated with a lower risk of pregnancy complications, as well as with improved maternal metabolic, cardiovascular, and mental health [18,35,52,53]. The postpartum period was characterized by lower erythrocyte DHA concentrations compared with pregnancy, which may reflect continued maternal DHA depletion through secretion into breast milk during lactation [54].

The FA composition of RBC is determined not only by diet but also by genetic variants of the FA desaturase (FADS) cluster (FADS1, FADS2) and elongase genes, which modulate FA desaturation and elongation; age, ethnicity, and health status serve as additional modifiers. The role of gene–diet interactions remains poorly understood and was not evaluated in our study [12]. Studies in pregnant women most often examine the dietary intake of LCPUFAs *n*-3 and *n*-6 and changes in these FAs in blood plasma or erythrocyte membranes. In pregnant women, a moderate correlation was found between dietary EPA/DHA and their levels in plasma (r = 0.21) and erythrocytes (r = 0.37–0.61); for linoleic and arachidonic acids, the correlations were weak or insufficient [35,55,56]. This confirms the relevance of erythrocyte membrane phospholipids as a biomarker of EPA and DHA consumption, but not of other PUFAs. A similar significant relationship between dietary EPA/DHA and erythrocyte DHA has also been established. It was highlighted that significant negative correlation between EPA+DHA intake and AA in erythrocyte phospholipids can be observed [57]. Docosapentaenoic acid (DPA) supplementation modified the total FA composition of tissues, increasing the content of DPA, EPA, and DHA, with a progressive effect on EPA [57]. Additionally, stearidonic acid is an omega-3 FA that demonstrates superior bioconversion to EPA compared with ALA by circumventing the Δ6-desaturase-catalyzed rate-limiting step, the primary enzymatic constraint underlying the characteristically low conversion efficiency of ALA in humans [58,59,60]. Negative correlations between dietary and erythrocyte PUFAs may be partly explained by the fact that palmitoleic acid is synthesized predominantly during de novo lipogenesis via stearoyl CoA desaturase 1 (SCD1), a key enzyme in the synthesis of oleate (18:1c9) and cis-palmitoleate (16:1c9), primarily in the liver [61]. In addition to the cis-isomer, trans-palmitoleate can be formed endogenously by shortening the vaccenic acid chain (18:1t11) with an efficiency of ~17% [61]. SCD1 activity is enhanced by dietary carbohydrates and proteins, whereas PUFAs suppress de novo lipogenesis by decreasing the expression of acetyl-CoA carboxylase alpha, FA synthase, and SCD through inhibition of sterol regulatory element-binding protein 1 (SREBP-1) [62], as well as through SREBP1-c and peroxisome proliferator-activated receptor-α, the latter of which further modulates β-oxidation [63,64]. The decrease in 20:2 in erythrocytes upon intake of 14:0–17:0 could be due to the convergence of several mechanisms, including the inhibition of elongation of very-long-chain FA protein 5 (ELOVL5) and SCD-1, suppression of FADS2/ELOVL5 at the transcriptional level, redistribution of acyl-CoA, and membrane remodeling via the Lands cycle [65,66,67,68]. Although all correlations between dietary and erythrocyte FAs were nonsignificant after Benjamini–Hochberg correction, this attenuation may reflect several factors: reduced statistical power under stringent multiple-comparison correction, measurement error, and homeostatic regulation of membrane composition.

Consumption of odd-chain FAs (15:0, 17:0), which make up only 1% and 0.5% of the FAs in whole milk, respectively, is associated with a reduced risk of cardiovascular disease, type 2 diabetes mellitus, and obesity [67]. Dietary 15:0 intake is inversely correlated with the activity of liver enzymes (alanine aminotransferase, aspartate aminotransferase, and gamma-glutamyl transferase), accumulation of fat in the liver, and glucose levels [64,69,70]; a significant increase in its levels in the blood is achieved only with the consumption of whole milk products [70]. Our study showed a weak but statistically significant correlation between dietary and erythrocyte 15:0 that lost statistical significance after correction. Both markers, 15:0 and trans-16:1 *n*-7, are useful as biomarkers of milk fat consumption, but the low specificity of 15:0 and 17:0 calls for caution in epidemiological interpretations [71,72,73]. The correlation between milk fat intake and the FA composition of blood lipids can range from poor to good (r = 0.23–0.62), especially for the characteristic milk FAs—14:0, 14:1, 15:0, and 17:1 [74]. 14:1, 15:0, and 17:1 are co-secreted in the fat of ruminants; 17:1 is further linked to 15:0 via the metabolic precursor 17:0 through chain-shortening β-oxidation [74,75]. The degree of FA oxidation decreases with increasing carbon chain length [75].

In addition to dietary fat, other macronutrients can also contribute to the FA composition of RBC. Dietary protein can indirectly modulate this composition through insulin signaling, SREBP-1 regulation, and the production of branched-chain FAs by the gut microbiota [76,77,78]. Dietary fiber, by increasing the synthesis of short-chain FAs, can also influence lipid metabolism [79]. Although the negative correlation between RBC 20:2, *n*-6 and dietary vitamins A, K, and C did not remain statistically significant after correction, this observed trend can be partially explained by the fact that foods rich in these vitamins are typically low in linoleic acid, the precursor of 20:2, *n*-6, which reduces its availability for incorporation into RBC membranes and theoretically increases the bioavailability of omega-3 PUFAs [8]. In addition, vitamin A regulates the expression of lipid metabolism genes via the nuclear receptors RAR/RXR [80], and RXR activation by retinoids can influence the transcription of the FADS2 gene, potentially modulating the synthesis of LCPUFA [81]. The relationship between vitamin E and FA profiles may reflect common dietary sources—oils and nuts rich in both PUFAs and tocopherols [82]. The negative correlation between 22:5 *n*-6 and dietary vitamin E, which likewise did not survive correction, is currently not sufficiently supported by the literature. Data on the relationship between oxidation indices and dietary factors are limited [45,83]. These associations should be interpreted with caution given the loss of statistical significance after Benjamini–Hochberg correction for multiple comparisons.

A positive correlation was initially found between dietary erucic acid intake and SEPP1 levels. On the one hand, erucic acid has demonstrated neuroprotective and anti-inflammatory effects, but at high concentrations it is associated with the development of liver steatosis and metabolic disorders [84,85]; MDA, a product of lipid peroxidation, is a widely accepted biomarker of oxidative stress [86]. The negative associations found between MDA and SFA consumption may indicate their lower susceptibility to oxidation [87]; nevertheless, given the loss of significance after adjustment, these findings should be interpreted with caution. The detected correlation between 18:3, *n*-3 and FRAP was the only association to remain significant after correction for multiple testing; other FA–oxidative stress correlations were nominally significant but not robust to subgroup analysis or multiplicity correction. *N*-3 LCPUFAs are known to exert antioxidant and anti-inflammatory properties and may thereby reduce oxidative stress [88]. Several gestational complications have been linked to lower erythrocyte *n*-3 FA levels, supporting a role for these FAs in their pathophysiology [89,90,91].

Our study identified statistically significant differences in erythrocyte SFA (14:0, 18:0) and PUFA (22:5, *n*-3, 18:2, *n*-6, 20:3, *n*-6, 22:4, *n*-6) levels according to smoking status. Previous studies have shown that maternal smoking is associated with reduced DHA and EPA content in maternal blood, together with higher total SFA, 18:2, *n*-6, and 20:4, *n*-6 concentrations and an elevated *n*-6/*n*-3 ratio in smokers. Smoking may impair the conversion of 18:3, *n*-3 to 22:6, *n*-3 and alter the conversion rate of *n*-6 FAs to eicosanoids. However, further research is needed, as it remains difficult to disentangle changes in FA metabolism and synthesis, oxidative degradation of unsaturated FAs following cigarette smoke exposure, and the concurrent influence of dietary intake [49,92]. Mean erythrocyte levels of 15:0, 20:0, 16:1, 18:3, *n*-3, 20:2, *n*-6, and 22:5, *n*-6 also differed by alcohol consumption status. Most studies report that alcohol intake before and in early pregnancy is associated with lower *n*-3 PUFA concentrations, including 22:6, *n*-3, 20:5, *n*-3, and 22:5, *n*-3. These findings suggest that the effect of alcohol on PUFA status may depend on the amount, frequency, and type of alcohol consumed [93].

Our study found a statistically significant correlation between maternal age and MDA levels. Another study showed that an increase in the IQR of 1-hydroxypyrene (65.8 pg/mL) was associated with a 7.73% (95% CI: 3.18%, 12.3%) increase in MDA levels during pregnancy, particularly in the first and second trimesters, after accounting for maternal age, lifestyle, and socioeconomic factors [94]. Additionally, a statistically significant positive correlation was observed between BMI and the level of MUFAs in RBC. Pre-gestational BMI may be related to the lipid composition of RBC membranes, and similar results were obtained in an Italian population, though this association had not previously been detected in a Latvian sample [95,96].

While unadjusted analyses suggested possible associations between FRAP and newborn anthropometric measures, none of these associations remained statistically significant after correction for multiple comparisons, indicating that—at least in this sample size—there is no robust evidence linking these maternal biomarkers to neonatal anthropometric outcomes; the nominal signals observed should be considered exploratory and hypothesis-generating rather than confirmatory. Limited data have been reported in the literature, mainly focusing on associations between erythrocyte FAs and anthropometric measures in newborns—higher *n*-6 levels may be associated with low birth weight, and it has been noted that maternal erythrocyte PUFAs may be associated with offspring body weight up to 2 years of age [51,97].

The strengths of this study include the use of a validated food frequency questionnaire and RBCs as a reliable tool for the long-term assessment of FA intake. Several limitations of the present study should be acknowledged. First, the relatively small sample size limits both the statistical power and the generalizability of the findings to broader populations. Subgroup-level correlations should therefore be interpreted as underpowered and exploratory, viewed as hypothesis-generating rather than confirmatory, and should be replicated in larger, adequately powered samples before firm conclusions are drawn. Second, the cross-sectional design precludes the establishment of causal relationships between the variables examined. Furthermore, the results do not fully account for dietary patterns, supplement use, and endogenous factors such as FA metabolism, genetics, and comorbidities, all of which may act as important confounders and warrant consideration in future research. Although all samples were handled under standardized temperature-controlled conditions and stored at −80 °C prior to analysis, a potential influence of long-term storage on MDA levels cannot be fully excluded. Therefore, the observed MDA concentrations may be partially affected by pre-analytical storage conditions. It should also be noted that after applying corrections for multiple comparisons, only some of the correlations identified in the analyses of associations between laboratory parameters, dietary and lifestyle factors, and maternal and neonatal outcomes remained statistically significant, suggesting limited robustness of the observed associations. Accordingly, these findings should be interpreted with caution and require validation in independent studies.

Data on FA levels during pregnancy and the factors influencing them remain limited. However, maternal RBC FAs have potential as prognostic biomarkers for maternal and fetal health, as well as for the early detection of pregnancy complications. Nonetheless, a wide range of factors must be considered, including diet, lifestyle, genetics, body composition, and oxidative stress.

## 5. Conclusions

This study’s participants had high fat intakes, with EPA and DHA intakes in line with recommendations. The lack of reference values for FAs and oxidative stress markers in pregnant women complicates the interpretation of the results.

Given the small sample size, cross-sectional design, and large number of associations tested, this study should be regarded as exploratory. Nonetheless, several associations remained statistically significant after correction for multiple comparisons and can be considered statistically supported: erythrocyte 18:3, *n*-3 was correlated with FRAP, alcohol consumption and smoking history were associated with alterations in erythrocyte FA profiles, maternal age was related to plasma MDA concentration, and pre-gestational BMI was associated with MUFA levels. All other associations observed in preliminary analyses did not survive correction for multiple testing and should be interpreted as hypothesis-generating rather than confirmatory, warranting replication in larger, adequately powered studies.

A more in-depth study of the mechanisms underlying the relationships between RBC FAs, oxidative stress, and the factors that modulate them appears to be essential for the development of valid prognostic and diagnostic biomarkers aimed at monitoring maternal and fetal health and preventing obstetric complications.

## Figures and Tables

**Table 1:** Baseline characteristics of study participants.

Variable	*n* (%)
Participants	
Pregnant women	38 (51.4)
7-day postpartum women	36 (48.6)
Nationality	
Latvian	54 (73.0)
Russian	15 (20.3)
Other	1 (1.4)
Missing	4 (5.4)
Marital status	
Married	50 (67.6)
Live in partnership	21 (28.4)
Single mother	2 (2.7)
Missing	1 (1.4)
Education	
Primary education/incomplete secondary education	4 (5.4)
General special/secondary/vocational education	15 (20.3)
Higher education	47 (63.5)
Incomplete higher education	8 (10.8)
Pre-pregnancy BMI group	
Underweight	2 (2.7)
Normal weight	45 (60.8)
Overweight	16 (21.6)
Obese	7 (9.5)
Missing	4 (5.4)
Gestational complications	
None	52 (70.3)
Labor induction	6 (8.1)
Gestational hypertension	1 (1.4)
Gestational diabetes	4 (5.4)
Preterm birth	2 (2.7)
Large-for-gestational-age infant	6 (8.1)
Anemia	6 (8.1)
Urinary tract infection	4 (5.4)
Time spent walking and cycling per day during pregnancy	
<15 min	1 (1.4)
15–30 min	18 (24.3)
30–60 min	32 (43.2)
>60 min	22 (29.7)
Missing	1 (1.4)
Frequency of at least 30 min of physical exercise during pregnancy	
Daily	2 (2.7)
4–6 times a week	4 (5.4)
2–3 times a week	17 (23.0)
Once a week	14 (18.9)
2–3 times a month	7 (9.5)
Some occurrences annually or less frequently or never	29 (39.2)
Cannot exercise due to illness or disability	1 (1.4)
Alcohol consumption during pregnancy	
Yes	10 (13.5)
No	49 (66.2)
Did not consume it after finding out about current pregnancy	13 (17.6)
Missing	2 (2.7)
Smoking during pregnancy	
Yes	3 (4.1)
No	67 (90.6)
Missing	4 (5.4)

BMI, body mass index = kg/m^2^.

**Table 2:** Newborns’ anthropometric measurements.

Variable	*n*	Mean ±SD
Weight (g)	36	3538.7 ± 728.7
Height (cm)	35	52.9 ± 4.4
Head circumference (cm)	35	34.7 ± 2.7
Chest circumference (cm)	35	34 ± 3.3

**Table 3:** Daily median intake (Q1–Q3) of energy and nutrients from foods.

Energy/Nutrient	Median (Q1–Q3)
Energy (kcal)	2226.1 (1630.8–2761.0)
Protein (g)	102.0 (72.0–121.1)
Protein (% of energy)	18.2 (15.8–20.4)
Carbohydrate (g)	215.5 (158.3–247.4)
Carbohydrate (% of energy)	37.8 (35.1–41.2)
Soluble fiber (g)	7.5 (5.3–9.4)
Insoluble fiber (g)	17 (12.5–20.5)
Fat (g)	104.0 (74.6–124.8)
Fat (% of energy)	42.2 (39.0–45.3)
Fructose (g)	24.6 (14.8–30.5)
SFAs (g)	16.7 (12.0–23.0)
4:0	0.9 (0.6–1.3)
6:0	0.6 (0.4–0.8)
8:0	0.4 (0.3–0.5)
10:0	0.8 (0.5–1.1)
12:0	1.2 (0.8–1.6)
14:0	3.4 (2.2–4.8)
15:0	0.3 (0.2–0.5)
16:0	17.0 (12.3–22.5)
17:0	0.3 (0.2–0.4)
18:0	6.4 (4.7–9.2)
20:0	0.3 (0.3–0.4)
22:0	n/a
24:0	n/a
MUFAs (g)	53.3 (40.0–68.5)
10:1	0.2 (0.1–0.2)
14:1	0.4 (0.3–0.6)
16:1	1.9 (1.5–2.7)
17:1	0.3 (0.2–0.4)
18:1, *n*-9	32.8 (24.4–42.8)
20:1	0.5 (0.4–0.7)
22:1	0.2 (0.1–0.3)
24:1, *n*-9	n/a
PUFAs (g)	16.7 (12.0–22.1)
*n*-3 FAs	2.7 (2.1–4.2)
18:3, *n*-3	2.2 (1.7–3.3)
18:4, *n*-3	n/a
20:5, *n*-3	0.2 (0.1–0.3)
22:3, *n*-3	n/a
22:5, *n*-3	n/a
22:6, *n*-3	0.2 (0.1–0.5)
*n*-6 FAs	13.0 (9.2–17.7)
16:2 *n*-6	n/a
18:2, *n*-6	12.8 (8.9–17.3)
20:2, *n*-6	n/a
20:3, *n*-6	n/a
20:4, *n*-6	0.2 (0.1–0.3)
22:4, *n*-6	n/a
Vitamin A (µg)	1604 (1192.8–2184.1)
Vitamin D (µg)	1.8 (1.2–3.4)
Vitamin K (µg)	360.3 (282.0–450.1)
Vitamin E (mg) as tocopherol equivalent	13.4 (9.6–19.8)
Vitamin E (mg) as α-tocopherol	12.3 (8.7–18.1)
Vitamin C (mg)	126.9 (83.3–192.4)
Iron (mg)	12.5 (10.2–17.8)

SFAs—saturated fatty acids; MUFAs—monounsaturated fatty acids; PUFAs—polyunsaturated fatty acids; FAs—fatty acids; n/a—not available.

**Table 4:** Daily median FA (g) intake (Q1–Q3) by food group.

Food Group	SFAs	MUFAs	PUFAs	*n*-3	*n*-6
Jam	0.7 (0.2–1.5)	2.9 (0.5–3.6)	0.1 (0.0–0.3)	n/a	0.1 (0.0–0.3)
Milk and milk products	5.0 (3.4–8.3)	8.3 (5.8–13.8)	0.4 (0.3–0.6)	0.2 (0.1–0.3)	0.4 (0.3–0.6)
Fats and oils	0.6 (0.4–1.1)	10.1 (5.4–12.2)	1.5 (1.0–2.7)	0.1 (0.1–0.3)	1.5 (1.0–2.7)
Meat and meat products	1.0 (0.5–1.9)	5.6 (2.8–10.0)	1.5 (0.9–2.1)	0.2 (0.1–0.2)	1.4 (0.9–2.1)
Soups	0.7 (0.3–1.1)	2.4 (1.2–4.7)	0.5 (0.3–1.1)	0.1 (0.0–0.2)	0.5 (0.3–1.1)
Fish	0.3 (0.1–0.5)	0.8 (0.3–1.7)	0.3 (0.1–0.6)	0.2 (0.1–0.5)	0.1 (0.0–0.2)
Eggs	0.3 (0.1–0.4)	2 (0.9–3.1)	0.4 (0.2–0.6)	0.1 (0.1–0.2)	0.3 (0.2–0.5)
Bread and pastries	0.1 (0.0–0.1)	0.4 (0.2–0.9)	0.4 (0.2–0.7)	n/a	0.4 (0.2–0.7)
Potatoes	0.3 (0.2–0.6)	1.4 (0.7–2.7)	0.8 (0.3–1.8)	0.2 (0.1–0.5)	0.8 (0.3–1.8)
Vegetables	n/a	1.3 (0.5–3.4)	0.3 (0.2–0.5)	0.1 (0.1–0.2)	0.3 (0.2–0.5)
Pancakes	0.2 (0.1–0.5)	0.9 (0.4–1.9)	0.1 (0.1–0.2)	n/a	0.1 (0.1–0.2)
Nuts and seeds	0.1 (0.0–0.4)	2.3 (0.7–7.1)	1.3 (0.3–3.5)	0.2 (0.0–0.6)	1.3 (0.3–3.5)
Sweets	1.8 (0.9–3.1)	4.9 (2.2–7.5)	1.0 (0.5–2.3)	0.2 (0.1–0.5)	1.0 (0.5–2.3)

SFAs—saturated fatty acids; MUFAs—monounsaturated fatty acids; PUFAs—polyunsaturated fatty acids; FAs—fatty acids; n/a—not available.

**Table 5:** Daily intake of nutrients from food supplements.

Nutrient	*n* (%)	Mean ± SD
Vitamin A (µg)	3 (4.1)	n/a
Vitamin D (µg)	64 (86.5)	63.3 ± 48.0
Vitamin E (mg) as tocopherol equivalent	3 (4.1)	n/a
Vitamin E (mg) as α-tocopherol	1 (1.4)	n/a
Vitamin C (mg)	2 (2.7)	n/a
Iron (mg)	68 (91.9)	38.1 ± 31.8
20:5, *n*-3 (mg)	31 (41.9)	297.7 ± 228.2
22:6, *n*-3 (mg)	31 (41.9)	198.5 ± 152.1

n/a—not available.

**Table 6:** FA composition (% of total FAs) and differences in erythrocyte phospholipids in pregnant and postpartum women.

FA	Mean ± SD	Median (Q1–Q3)	Pregnant Women (*n* = 38)	Postpartum Women (*n* = 36)	*p* Value ^a,b^
			Median (Q1–Q3)	Median (Q1–Q3)	
SFAs	-	55.7 (49.8–65.3)	55.6 (51.9–61.3)	57.4 (48.9–65.8)	0.016 *
14:0	1.1 ± 1.0	-	1.0 (0.7–1.2)	1.0 (0.7–1.2)	0.913
15:0	0.6 ± 0.3	-	0.5 (0.4–0.7)	0.6 (0.5–0.9)	0.112
16:0	-	30.5 (28.7–37.5)	30.0 (28.8–37.0)	32.4 (28.4–38.0)	0.944
18:0	18.6 ± 4.0	-	17.4 (16.2–22.0)	17.8 (14.4–21.6)	0.858
20:0	-	0.6 (0.5–0.7)	0.6 (0.5–0.7)	0.6 (0.5–0.8)	0.089
22:0	-	0.9 (0.7–1.1)	0.9 (0.8–1.1)	0.9 (0.7–1.1)	0.810
24:0	-	0.7 (0.5–1.0)	0.7 (0.5–1.0)	0.6 (0.5–0.9)	0.393
MUFAs	15.9 ± 3.2	-	16.4 (14.0–19.0)	16.3 (13.0–18.1)	0.333
16:1	-	0.7 (0.5–0.9)	0.6 (0.5–0.9)	0.7 (0.5–1.0)	0.359
18:1, *n*-9	12.8 ± 3.0	-	13.6 (10.7–15.2)	13.2 (10.2–14.7)	0.378
20:1	-	0.8 (0.6–1.0)	0.8 (0.6–0.9)	0.7 (0.6–1.0)	0.935
24:1, *n*-9	-	2.3 (1.7–2.9)	2.4 (1.8–2.9)	2.3 (1.6–2.9)	0.413
PUFAs	19.5 ± 4.7	-	20.0 (16.2–22.5)	19.4 (15.6–24.0)	0.870
*n*-3 FAs	7.2 ± 2.1	-	7.6 (5.8–9.7)	6.9 (5.6–8.1)	0.072
18:3, *n*-3	-	0.6 (0.4–0.8)	0.5 (0.4–0.8)	0.7 (0.5–0.9)	0.051
20:5, *n*-3	-	1.8 (1.4–2.3)	1.9 (1.4–2.4)	1.7 (1.4–2.3)	0.288
22:5, *n*-3	-	1.2 (1.0–1.6)	1.2 (0.9–1.6)	1.2 (1.1–1.6)	0.851
22:6, *n*-3	-	2.9 (1.9–3.7)	3.1 (2.2–4.3)	2.7 (1.6–3.4)	0.027 *
*n*-6 FAs	14.8 ± 5.0	-	15.4 (11.5–17.8)	14.9 (11.0–19.8)	0.200
18:3, *n*-6	-	0.7 (0.4–0.9)	0.7 (0.3–0.9)	0.8 (0.5–1.1)	0.080
18:2, *n*-6	-	7.5 (5.6–9.1)	7.6 (6.1–8.9)	7.3 (5.5–9.6)	0.922
20:2, *n*-6	-	0.5 (0.4–0.8)	0.5 (0.4–0.8)	0.6 (0.5–0.8)	0.029 *
20:3, *n*-6	-	1.1 (0.8–1.4)	1.1 (0.8–1.3)	1.2 (0.9–1.6)	0.261
20:4, *n*-6	-	5.8 (4.3–7.4)	5.8 (3.9–7.3)	5.9 (4.4–7.5)	0.721
22:4, *n*-6	-	1.7 (1.2–2.0)	1.6 (1.0–2.1)	1.7 (1.3–2.0)	0.570
22:5, *n*-6	-	0.7 (0.5–0.9)	0.7 (0.5–0.8)	0.8 (0.5–0.9)	0.166

^a^ Mann–Whitney U-test and ^b^ independent samples *t*-test used to compare medians between groups. * *p* < 0.05. SFAs—saturated fatty acids; MUFAs—monounsaturated fatty acids; PUFAs—polyunsaturated fatty acids; FAs—fatty acids.

**Table 7:** Oxidative stress and antioxidant status markers and differences in parameters in pregnant and postpartum women.

Parameter	*n* (%)	Median (Q1–Q3)	Pregnant Women (*n* = 38)	Postpartum Women (*n* = 36)	*p* Value ^a,b^
			Median (Q1–Q3)	Median (Q1–Q3)	
SEPP1 (mg/L)	74 (100)	10.2 (7.9–14.1)	10.2 (7.1–13.9)	10.3 (8.1–14.2)	0.459
MDA (mmol/L)	74 (100)	4.0 (2.7–5.3)	4.0 (2.9–4.9)	4.0 (2.6–5.5)	0.974
FRAP (Fe^2+^ mmol/L)	72 (97.3)	0.7 (0.6–0.8)	0.7 (0.5–0.8)	0.7 (0.6–0.8)	0.310
GPx (U/mL)	72 (97.3)	7.1 (5.9–8.1)	6.9 (5.7–8.1)	7.5 (5.9–8.4)	0.469

^a^ Mann–Whitney U-test and ^b^ independent samples *t*-test used to compare medians between groups. SEPP1—selenoprotein P; MDA—malondialdehyde; FRAP—Ferric Reducing Antioxidant Power; GPx—glutathione peroxidase.

**Table 8:** Correlations between nutrients and markers of oxidative stress and antioxidant status.

Nutrient	SEPP1 (mg/L)			MDA (mmol/L)			FRAP (Fe^2+^ mmol/L)			GPx (U/mL)		
	r_s_	*p*	*p*_adj_	r_s_	*p*	*p*_adj_	r_s_	*p*	*p*_adj_	r_s_	*p*	*p*_adj_
Fructose (g)	−0.199	0.088	0.354	−0.199	0.089	0.375	−0.208	0.079	0.316	−0.231	0.047	0.189
Soluble fiber (g)	−0.114	0.334	1.000	−0.092	0.437	1.000	−0.239	0.043	0.174	−0.217	0.063	0.253
Insoluble fiber (g)	−0.100	0.399	1.000	−0.050	0.670	1.000	−0.201	0.091	0.364	−0.229	0.049	0.197
Vitamin A (µg)	−0.168	0.153	0.610	−0.094	0.428	1.000	0.025	0.838	1.000	0.039	0.740	1.000
Vitamin D (µg)	0.051	0.665	1.000	0.100	0.397	1.000	−0.102	0.395	1.000	−0.173	0.140	0.559
Vitamin E (mg) as tocopherol equivalent	0.004	0.973	1.000	−0.060	0.613	1.000	−0.178	0.134	0.536	−0.198	0.091	0.364
Vitamin E (mg) as α-tocopherol	0.016	0.891	1.000	−0.054	0.649	1.000	−0.180	0.131	0.523	−0.193	0.100	0.399
Vitamin K (μg)	−0.155	0.187	0.747	−0.050	0.673	1.000	−0.057	0.634	1.000	−0.092	0.433	1.000
Vitamin C (mg)	−0.249	0.033	0.131	−0.091	0.438	1.000	−0.080	0.506	1.000	−0.126	0.283	1.000
Iron (mg)	−0.084	0.476	1.000	−0.174	0.138	0.550	0.026	0.829	1.000	−0.006	0.959	1.000

r_s_ = Spearman’s rank correlation coefficient; *p*_adj_—*p* adjusted; SEPP1—selenoprotein P; MDA—malondialdehyde; FRAP—Ferric Reducing Antioxidant Power; GPx—glutathione peroxidase.

**Table 9:** Correlation between erythrocyte FAs and markers of oxidative stress and antioxidant status.

Erythrocyte FA	SEPP1 (mg/L)			MDA (mmol/L)			FRAP (Fe^2+^ mmol/L)			GPx (U/mL)		
	r_s_	*p*	*p*_adj_	r_s_	*p*	*p*_adj_	r_s_	*p*	*p*_adj_	r_s_	*p*	*p*_adj_
SFAs	−0.072	0.543	0.091	−0.198	0.091	0.364	0.114	0.342	1.000	−0.141	0.230	0.919
14:0	0.036	0.761	1.000	−0.148	0.208	0.832	0.060	0.616	1.000	−0.130	0.268	1.000
15:0	0.138	0.242	0.970	−0.023	0.848	1.000	0.211	0.075	0.298	−0.001	0.995	1.000
16:0	−0.108	0.358	1.000	−0.103	0.380	1.000	0.096	0.422	1.000	−0.134	0.253	1.000
18:0	−0.070	0.554	1.000	−0.220	0.060	0.906	0.014	0.906	1.000	−0.126	0.286	1.000
20:0	0.168	0.152	0.609	−0.034	0.770	1.000	0.192	0.106	0.425	0.114	0.332	1.000
22:0	−0.013	0.912	1.000	−0.049	0.681	1.000	0.046	0.703	1.000	0.053	0.656	1.000
24:0	0.209	0.074	0.297	0.062	0.600	1.000	0.099	0.408	1.000	0.080	0.500	1.000
MUFAs	−0.070	0.555	1.000	0.098	0.404	1.000	−0.064	0.593	1.000	0.116	0.324	1.000
16:1	0.151	0.200	0.799	0.114	0.334	1.000	0.258	0.029	0.116	0.149	0.205	0.819
18:1, *n*-9	−0.074	0.529	1.000	0.106	0.370	1.000	−0.065	0.588	1.000	0.132	0.263	1.000
20:1	0.062	0.598	1.000	0.144	0.222	0.888	0.095	0.429	1.000	−0.071	0.550	1.000
24:1, *n*-9	0.023	0.847	1.000	0.006	0.957	1.000	0.006	0.957	1.000	n/a	n/a	n/a
PUFAs	0.119	0.314	1.000	0.157	0.182	0.728	−0.106	0.377	1.000	0.111	0.347	1.000
*n*-3 FAs	0.064	0.588	1.000	0.128	0.277	1.000	−0.045	0.706	1.000	0.075	0.526	1.000
18:3, *n*-3	0.095	0.423	1.000	−0.129	0.275	1.000	0.359	0.002	0.008	0.232	0.047	0.187
20:5, *n*-3	−0.035	0.766	1.000	−0.017	0.888	1.000	−0.022	0.857	1.000	0.090	0.447	1.000
22:5, *n*-3	0.080	0.498	0.602	0.062	0.602	1.000	0.016	0.896	1.000	0.016	0.892	1.000
22:6, *n*-3	0.095	0.422	1.000	0.221	0.058	0.233	−0.131	0.272	1.000	0.009	0.939	1.000
*n*-6 FAs	0.096	0.417	1.000	0.178	0.129	0.514	−0.150	0.208	0.831	0.070	0.555	1.000
18:3, *n*-6	−0.016	0.895	1.000	0.027	0.817	1.000	0.244	0.039	0.157	−0.042	0.723	1.000
18:2, *n*-6	0.035	0.770	1.000	0.139	0.237	0.948	−0.182	0.127	0.507	0.084	0.475	1.000
20:2, *n*-6	0.049	0.680	1.000	0.017	0.883	1.000	0.291	0.013	0.053	0.036	0.763	1.000
20:3, *n*-6	0.132	0.250	1.000	0.113	0.340	1.000	0.009	0.940	1.000	0.042	0.723	1.000
20:4, *n*-6	0.099	0.402	1.000	0.214	0.067	0.269	−0.105	0.379	1.000	0.023	0.846	1.000
22:4, *n*-6	0.102	0.390	1.000	0.227	0.052	0.209	−0.048	0.678	1.000	−0.013	0.912	1.000
22:5, *n*-6	0.156	0.185	0.740	0.004	0.971	1.000	0.180	0.131	0.523	0.043	0.717	1.000

r_s_ = Spearman’s rank correlation coefficient; *p*_adj_—*p* adjusted; SFAs—saturated fatty acids; MUFAs—monounsaturated fatty acids; PUFAs—polyunsaturated fatty acids; FAs—fatty acids; SEPP1—selenoprotein P; MDA—malondialdehyde; FRAP—Ferric Reducing Antioxidant Power; GPx—glutathione peroxidase; n/a—not available.

**Table 10:** Differences in FA composition (% of total FAs) in erythrocyte phospholipids in women with different lifestyle factors, presented as medians (Q1–Q3) ^a^.

Erythrocyte FA	Smoking History		*p* Value	Alcohol Consumption Status		*p* Value	Physical Activity > 30 min per day		*p* Value
	Yes (*n* = 52)	No (*n* = 22)		Yes (*n* = 10)	No (*n* = 49)		Yes (*n* = 19)	No (*n* = 54)	
SFAs	53.3 (48.9–62.7)	58.9 (55.2–66.5)	0.009	54.2 (47.0–58.1)	55.6 (50.0–65.9)	0.315	56.1 (50.2–66.0)	53.7 (49.6–61.8)	0.517
14:0	1.0 (0.6–1.1)	1.2 (0.8–1.3)	0.040	0.9 (0.6–1.4)	1.0 (0.7–1.1)	0.265	1.0 (0.7–1.2)	1.0 (0.7–1.2)	0.709
15:0	0.5 (0.4–0.8)	0.7 (0.4–0.7)	0.680	0.5 (0.4–0.6)	0.6 (0.5–0.8)	0.034	0.5 (0.4–0.8)	0.6 (0.4–0.7)	0.732
16:0	29.5 (28.4–36.6)	34.20 (29.0–39.2)	0.110	28.3 (27.4–35.7)	32.1 (28.9–38.8)	0.134	32.0 (28.7–38.2)	29.6 (28.3–36.3)	0.493
18:0	16.8 (14.6–21.2)	20.9 (17.5–22.6)	0.004	16.6 (14.2–19.3)	19.2 (15.9–21.6)	0.276	17.7 (15.6–22.1)	17.6 (15.9–21.4)	0.935
20:0	0.6 (0.5–0.8)	0.6 (0.5–0.7)	0.596	0.5 (0.4–0.5)	0.6 (0.5–0.8)	0.014	0.6 (0.5–0.7)	0.6 (0.5–0.8)	0.461
22:0	0.9 (0.7–1.1)	0.8 (0.7–0.9)	0.148	0.9 (0.6–1.2)	0.9 (0.8–1.1)	0.544	0.9 (0.7–1.1)	0.8 (0.7–1.0)	0.899
24:0	0.7 (0.5–1.1)	0.7 (0.4–0.9)	0.222	0.6 (0.5–1.0)	0.7 (0.5–0.9)	0.075	0.7 (0.5–1.1)	0.6 (0.5–0.9)	0.924
MUFAs	16.3 (13.2–18.3)	15.1 (13.1–19.1)	0.967	17.1 (16.1–18.9)	15.4 (12.9–18.7)	0.484	15.9 (12.9–18.9)	16.3 (14.7–18.7)	0.546
16:1	0.6 (0.5–0.9)	0.7 (0.5–0.9)	0.948	0.6 (0.5–0.8)	0.7 (0.6–0.9)	0.006	0.6 (0.5–0.9)	0.7 (0.5–0.9)	0.954
18:1	13.4 (10.5–14.8)	12.7 (10.1–16.0)	0.873	14.5 (13.5–15.1)	12.2 (10.1–15.1)	0.310	13.2 (10.1–15.0)	14.4 (11.8–15.6)	0.330
20:1	0.7 (0.6–1.0)	0.80 (0.7–1.0)	0.278	0.6 (0.4–0.8)	0.8 (0.6–1.1)	0.070	0.8 (0.6–1.0)	0.7 (0.6–0.9)	0.443
24:1	2.4 (1.6–2.9)	2.2 (1.8–2.6)	0.438	2.3 (1.6–2.9)	2.2 (1.7–2.9)	0.995	2.3 (1.7–2.9)	2.2 (1.5–2.9)	0.450
PUFAs	21.4 (17.2–24.5)	17.2 (14.9–20.9)	0.003	21.7 (18.1–24.2)	19.6 (16.0–22.8)	0.423	19.3 (16.2–23.3)	20.9 (15.6–22.5)	0.797
*n*-3	7.4 (6.1–8.9)	6.6 (5.4–7.9)	0.093	7.3 (6.2–9.3)	7.2 (5.7–8.7)	0.763	7.2 (5.6–8.9)	7.2 (5.8–8.5)	0.845
18:3, *n*-3	0.6 (0.4–0.9)	0.6 (0.6–0.8)	0.830	0.5 (0.3–0.7)	0.7 (0.5–0.9)	0.013	0.6 (0.4–0.8)	0.8 (0.4–0.9)	0.222
20:5, *n*-3	1.9 (1.4–2.4)	1.6 (1.3–2.0)	0.142	1.7 (1.3–2.1)	1.7 (1.4–2.3)	0.911	1.8 (1.4–2.3)	1.9 (1.4–1.3)	0.777
22:5, *n*-3	1.3 (1.1–1.7)	1.2 (0.9–1.4)	0.048	1.5 (1.1–1.8)	1.2 (1.0–1.5)	0.153	1.2 (1.0-1.6)	1.3 (1.2-1.5)	0.267
22:6, *n*-3	3.0 (2.1–3.9)	2.7 (1.5–3.2)	0.123	3.6 (2.1–4.7)	2.8 (1.9–3.7)	0.244	2.8 (1.8–3.7)	2.9 (2.3–3.8)	0.787
*n*-6	16.8 (12.5–19.3)	12.1 (9.8–15.1)	0.005	18.1 (15.0–19.9)	14.6 (11.0–18.8)	0.172	14.9 (10.8–18.6)	16.0 (11.2–18.6)	0.749
18:2, *n*-6	8.1 (6.2–9.7)	6.3 (5.0–7.5)	0.005	9.0 (7.2–10.1)	7.1 (5.3–9.1)	0.103	7.3 (5.5–9.1)	7.7 (5.6–8.7)	0.597
18:3, *n*-6	0.7 (0.4–1.0)	0.8 (0.4–0.9)	0.789	0.4 (0.4–0.7)	0.8 (0.6–1.0)	0.093	0.7 (0.4–1.0)	0.7 (0.5–0.9)	0.747
20:3, *n*-6	1.2 (0.9–1.5)	1.0 (0.8–1.1)	0.032	1.3 (0.8–1.5)	1.1 (0.7–1.4)	0.475	1.1 (0.8–1.4)	1.1 (0.9–1.4)	0.840
20:2, *n*-6	0.5 (0.4–0.7)	0.7 (0.4–0.9)	0.571	0.5 (0.4–0.5)	0.6 (0.5–0.1.0)	0.014	0.5 (0.4–0.8)	0.6 (0.4–0.8)	0.980
20:4, *n*-6	6.5 (4.6–7.6)	4.6 (2.5–6.0)	0.010	7.1 (6.3–7.7)	5.5 (4.1–7.3)	0.182	5.7 (4.2–7.3)	5.8 (4.3–7.6)	0.589
22:4, *n*-6	1.8 (1.2–2.1)	1.3 (1.0–1.7)	0.027	1.7 (1.1–2.2)	1.4 (1.1–2.1)	0.938	1.7 (1.1–2.0)	1.4 (1.3–2.3)	0.364
22:5, *n*-6	0.7 (0.5–0.9)	0.7 (0.5–0.8)	0.546	0.6 (0.5–0.9)	0.7 (0.5–0.9)	0.035	0.7 (0.5–0.9)	0.6 (0.5–0.8)	0.465

^a^ Mann–Whitney U-test used to compare medians between groups. SFAs—saturated fatty acids; MUFAs—monounsaturated fatty acids; PUFAs—polyunsaturated fatty acids; FA—fatty acid.

**Table 11:** Correlations between erythrocyte FA parameters and markers of oxidative stress and antioxidant status by anthropometric measurements of newborns.

Anthropometric Measurement		SFAs	MUFAs	PUFAs	*n*-3	*n*-6	SEPP1 (mg/L)	MDA (mmol/L)	FRAP (Fe^2+^ mmol/L)	GPx (U/mL)
Newborn weight (*n* = 36)	r_s_	0.328	−0.275	−0.339	−0.181	−0.393	−0.058	−0.146	0.411	−0.068
	95% CI	−0.010 to 0.598	−0.558 to 0.066	−0.607 to 0.002	−0.483 to 0.160	−0.647 to −0.062	−0.380 to 0.276	−0.454 to 0.194	0.076 to 0.663	−0.388 to 0.267
	*p*	0.051	0.105	0.043	0.291	0.018	0.737	0.396	0.014	0.693
	*p*_adj_	0.306	0.525	0.301	1.000	0.144	1.000	1.000	0.126	1.000
Newborn height (*n* = 35)	r_s_	0.224	−0.214	−0.272	−0.122	−0.299	−0.010	−0.023	0.347	−0.086
	95% CI	−0.123 to 0.521	−0.513 to 0.133	−0.559 to 0.073	−0.438 to 0.222	−0.580 to 0.046	−0.342 to 0.325	−0.353 to 0.313	0.000 to 0.620	−0.408 to 0.255
	*p*	0.196	0.218	0.114	0.486	0.081	0.956	0.897	0.044	0.623
	*p*_adj_	1.000	1.000	0.798	1.000	0.648	1.000	1.000	0.396	1.000
Newborn head circumference (*n* = 35)	r_s_	0.410	−0.389	−0.444	−0.288	−0.460	−0.144	−0.128	0.413	−0.186
	95% CI	0.075 to 0.662	−0.647 to −0.052	−0.686 to −0.114	−0.571 to 0.058	−0.697 to −0.132	−0.456 to 0.201	−0.444 to 0.215	0.072 to 0.667	−0.492 to 0.160
	*p*	0.014	0.021	0.008	0.094	0.005	0.411	0.462	0.015	0.284
	*p*_adj_	0.098	0.064	0.064	0.376	0.045	0.825	0.852	0.098	0.852
Newborn chest circumference (*n* = 35)	r_s_	0.359	−0.361	−0.365	−0.272	−0.387	−0.154	−0.197	0.372	−0.072
	95% CI	0.018 to 0.625	−0.626 to 0.020	−0.629 to −0.025	−0.559 to 0.074	−0.646 to −0.049	−0.465 to 0.191	−0.500 to 0.149	0.027 to 0.638	−0.397 to 0.268
	*p*	0.034	0.033	0.031	0.114	0.022	0.378	0.256	0.030	0.679
	*p*_adj_	0.217	0.217	0.217	0.240	0.198	0.456	0.768	0.240	0.768

r_s_ = Spearman’s rank correlation coefficient; CI—confidence interval; *p*_adj_—*p* adjusted; SFAs—saturated fatty acids; MUFAs—monounsaturated fatty acids; PUFAs—polyunsaturated fatty acids; FAs—fatty acids; SEPP1—selenoprotein P; MDA—malondialdehyde; FRAP—Ferric Reducing Antioxidant Power; GPx—glutathione peroxidase.

**Table 12:** Residual-based partial Spearman correlation coefficients between erythrocyte fatty acid parameters, markers of oxidative stress and antioxidant status, and neonatal anthropometric measurements, adjusted for gestational age, pre-pregnancy BMI, and maternal smoking.

Anthropometric Measurement	ESS		SFAs	MUFAs	PUFAs	*n*-3	*n*-6	SEPP1 (mg/L)	MDA (mmol/L)	FRAP (Fe^2+^ mmol/L)	GPx (U/mL)
Newborn weight (*n* = 36)	33	r	0.220	−0.167	−0.271	−0.012	−0.312	0.112	−0.098	0.369	−0.112
		95% CI	−0.132 to 0.522	−0.480 to 0.183	−0.582 to 0.080	−0.349 to 0.327	−0.594 to 0.037	−0.236 to 0.435	−0.423 to 0.249	0.017 to 0.639	−0.434 to 0.236
		*p*	0.212	0.344	0.121	0.946	0.072	0.528	0.580	0.035	0.529
		*p*_adj_	0.477	0.619	0.363	0.946	0.324	0.653	0.653	0.315	0.653
Newborn height (*n* = 35)	32	r	0.088	−0.024	−0.115	−0.046	−0.110	0.134	0.127	0.247	−0.085
		95% CI	−0.264 to 0.419	−0.365 to 0.322	−0.442 to 0.239	−0.383 to 0.302	−0.438 to 0.244	−0.221 to 0.457	0.482 to −0.228	−0.116 to 0.553	0.637 to 0.266
		*p*	0.628	0.893	0.524	0.800	0.542	0.458	0.482	0.172	0.637
		*p*_adj_	0.819	0.893	0.819	0.893	0.819	0.819	0.819	0.819	0.819
Newborn head circumference (*n* = 35)	32	r	0.205	−0.186	−0.265	−0.105	−0.223	0.088	0.089	0.477	−0.033
		95% CI	−0.153 to 0.515	−0.500 to 0.171	−0.562 to 0.092	−0.433 to 0.248	−0.529 to 0.135	−0.264 to 0.419	−0.263 to 0.420	0.134 to 0.718	−0.372 to 0.314
		*p*	0.254	0.300	0.136	0.561	0.212	0.628	0.624	0.006	0.855
		*p*_adj_	0.540	0.540	0.540	0.77	0.540	0.707	0.707	0.054	0.855
Newborn chest circumference (*n* = 35)	32	r	0.251	−0.287	−0.230	−0.155	−0.227	0.006	−0.091	0.309	0.045
		95% CI	−0.107 to 0.551	−0.579 to 0.069	−0.534 to 0.128	−0.475 to 0.201	−0.532 to 0.131	−0.338 to 0.349	−0.421 to 0.262	−0.053 to 0.599	−0.303 to 0.383
		*p*	0.159	0.105	0.199	0.388	0.204	0.972	0.616	0.085	0.802
		*p*_adj_	0.367	0.367	0.367	0.582	0.367	0.972	0.792	0.367	0.902

ESS—effective sample size; r = residual-based partial Spearman correlation coefficient; CI—confidence interval; *p*_adj_—*p* adjusted; SFAs—saturated fatty acids; MUFAs—monounsaturated fatty acids; PUFAs—polyunsaturated fatty acids; FAs—fatty acids; SEPP1—selenoprotein P; MDA—malondialdehyde; FRAP—Ferric Reducing Antioxidant Power; GPx—glutathione peroxidase.

## Data Availability

The original contributions presented in this study are included in the article. Further inquiries can be directed to the corresponding author.

## Abbreviations

The following abbreviations are used in this manuscript:

BMI	Body mass index
DHA	Docosahexaenoic acid
DOHaD	Developmental Origins of Health and Disease
DPA	Docosapentaenoic acid
EPA	Eicosapentaenoic acid
ELOVL5	Elongation of very-long-chain fatty acid protein 5
FA	Fatty acid
FADS	Fatty acid desaturase
FRAP	Ferric Reducing Antioxidant Power
FFQ	Food frequency questionnaire
GC-FID	Gas chromatography with flame ionization detection (GC-FID)
GPx	Glutathione peroxidase
LCPUFA	Long-chain polyunsaturated fatty acids
MDA	Malondialdehyde
MUFA	Monounsaturated fatty acid
PUFA	Polyunsaturated fatty acid
RAR	Retinoic acid receptor
RXR	Retinoic X receptor
RBC	Red blood cell
ROS	Reactive oxygen species
SCD	Stearoyl-CoA desaturase
SEPP1	Selenoprotein P
SREBP-1	Sterol regulatory element-binding protein 1
SFA	Saturated fatty acid
TBARS	Thiobarbituric acid reactive substances
